# Combined small cell lung carcinoma and giant cell carcinoma: a case report

**DOI:** 10.1186/s40792-017-0328-9

**Published:** 2017-03-31

**Authors:** Tomohito Saito, Koji Tsuta, Kento J. Fukumoto, Hiroshi Matsui, Toshifumi Konobu, Yoshitaro Torii, Takashi Yokoi, Takayasu Kurata, Hiroaki Kurokawa, Yoshiko Uemura, Yukihito Saito, Tomohiro Murakawa

**Affiliations:** 1 Department of Thoracic Surgery, Kansai Medical University Hospital, 2-3-1, Shinmachi, Hirakata City, Osaka Prefecture 573-1191 Japan; 2 Department of Pathology, Kansai Medical University Hospital, 2-3-1, Shinmachi, Hirakata City, Osaka Prefecture 573-1191 Japan; 3 Department of Thoracic Oncology, Kansai Medical University Hospital, 2-3-1, Shinmachi, Hirakata City, Osaka Prefecture 573-1191 Japan; 4 Department of Radiology, Kansai Medical University Hospital, 2-3-1, Shinmachi, Hirakata City, Osaka Prefecture 573-1191 Japan

**Keywords:** Combined small cell carcinoma, Giant cell carcinoma, Epithelial-to-mesenchymal transition

## Abstract

**Background:**

Combined small cell lung carcinoma (SCLC) is defined as SCLC combined with elements of non-small cell lung carcinoma (NSCLC), accounting for approximately 30% of cases of SCLC. However, combined SCLC and giant cell carcinoma (GC) is very rare.

**Case presentation:**

A 50-year-old woman with a 45 pack-year smoking history was referred to our hospital for further investigation of an abnormal left hilar shadow. Chest computed tomography (CT) revealed a 28-mm solid pulmonary nodule in the left lower lobe and an enlarged left hilar lymph node adjacent to the left main pulmonary artery. CT-guided biopsy of the pulmonary nodule led to the diagnosis of high-grade neuroendocrine carcinoma. The preoperative clinical stage was defined as cT1bN1M0. Thus, the patient underwent left lower lobectomy with ND2a-2 lymph node dissection via thoracotomy. Pathological investigation revealed a 22-mm tumor and dense sheet-like growth of small tumor cells with scant cytoplasm and finely granular nuclear chromatin. Moreover, there was a sheet-like growth of bizarre, highly pleomorphic mono- or occasionally multinucleated giant cells, accounting for approximately 40% of the tumor. Both the small and giant cell components were thyroid transcription factor-1-positive and p40-negative and exhibited neuroendocrine differentiation, as indicated by positivity for synaptophysin and CD56 and negativity for chromogranin A. While the small cell component was E-cadherin-positive and vimentin-negative, the giant cell component was E-cadherin-negative and vimentin-positive, indicating an epithelial-to-mesenchymal transition. Only the small cell component was found within the mediastinal and hilar lymph nodes. The final pathological diagnosis was combined SCLC and GC, pT1bN2M0, and pStage IIIA. The patient received adjuvant chemotherapy with 4 cycles of cisplatin and irinotecan. No sign of recurrence has been noted for 1 year after the surgery.

**Conclusions:**

This is the first detailed report of a unique case with combined SCLC and GC. The coexistence of SCLC and GC in the presented case might indicate several possibilities: (1) GC may arise from SCLC via epithelial-to-mesenchymal transition, (2) SCLC may arise from GC through phenotypic conversion, and (3) SCLC and GC may have derived from a common neuroendocrine origin. Further investigation is necessary to reveal the underlying pathological process.

## Background

Combined small cell lung carcinoma (SCLC) is defined as SCLC combined with elements of non-small cell lung carcinoma, and it accounts for approximately 30% of cases of SCLC [1]. However, SCLC combined with giant cell carcinoma (GC) is very rare. Nicholson et al. have reported the largest surgical case series of combined SCLC, showing that NSCLC components were found in 28 of 100 SCLCs: 16 with large cell carcinoma, 9 with adenocarcinoma, 3 with squamous cell carcinoma, but none with GC [2]. Fishback et al. found that only 1 of 48 cases of GC, which accounts for approximately 0.3% of all invasive lung malignancies, showed combined pathology with SCLC [3], for which detailed clinical, radiological, and pathological information were not reported. We report a case of combined SCLC and GC with preoperative medical images and findings of immunohistochemical staining.

## Case presentation

**Fig. 1:**
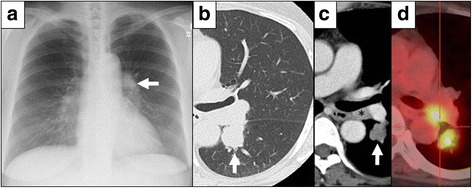
Medical imaging findings and gross appearance of the pulmonary nodule. **a** An abnormal left hilar shadow was observed (*white arrow*). **b**, **c** Contrast-enhanced computed tomography of the chest revealed a solid 28-mm pulmonary nodule at S6 of the left lower lobe (*white arrow*). **c** Hilar lymphadenopathy adjacent to the pulmonary artery was also found (*asterisk*). **d** FDG uptake was demonstrated in both the S6 nodule and the hilar lymph node

A 50-year-old woman with a 45 pack-year smoking history was referred to our hospital for further investigation of an abnormal left hilar shadow (Fig. 1a). The patient’s medical history included hypertension, and the patient had previously undergone an appendectomy at the age of 35 years and an abdominal myomectomy at the age of 49 years. The levels of tumor markers were as follows: carcinoembryonic antigen, 2.8 ng/mL; cytokeratin 19 fragment, 1.4 ng/mL; neuron-specific enolase, 21.0 ng/mL; and pro-gastrin-releasing peptide, 84.4 pg/mL.

Chest contrast-enhanced computed tomography (CT) revealed a 28-mm solid pulmonary nodule in the left lower lobe (S6) and an enlarged left hilar lymph node adjacent to the left main pulmonary artery (Fig. 1b, c). 18F-fluorodeoxyglucose (FDG) positron-emission tomography/CT showed increased FDG uptake in the S6 nodule and the hilar lymph node, in which the maximum standard uptake values were 5.3 and 5.0, respectively (Fig. 1d). CT-guided biopsy of the S6 nodule led to the pathological diagnosis of high-grade neuroendocrine carcinoma. Brain contrast-enhanced magnetic resonance imaging showed no signs of brain metastasis. Thus, the preoperative clinical stage was defined as cT1bN1M0. The patient underwent left lower lobectomy with ND2a-2 lymph node dissection via thoracotomy.

**Fig. 2:**
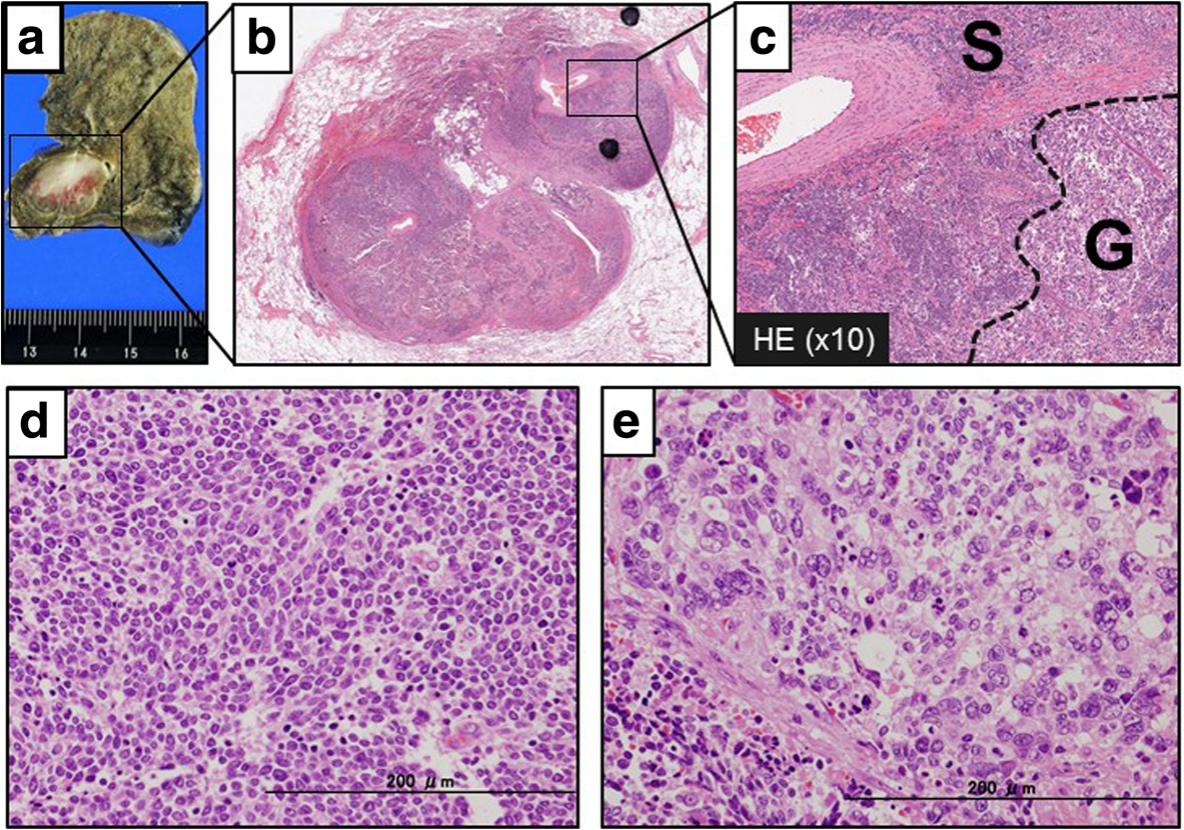
Macroscopic and microscopic findings of the pulmonary nodule. **a**, **b** A 22 × 18 × 12-mm nodule was found in the *left lower* lobe of the lung. **c**, **d** Pathological investigation revealed a dense sheet-like growth of small tumor cells with scant cytoplasm and finely granular nuclear chromatin. **e** There was also a sheet-like growth of bizarre, highly pleomorphic mono- or occasionally multinucleated giant cells. *HE*, hematoxylin and eosin, *G* giant cell component, *S* small cell component

**Fig. 3:**
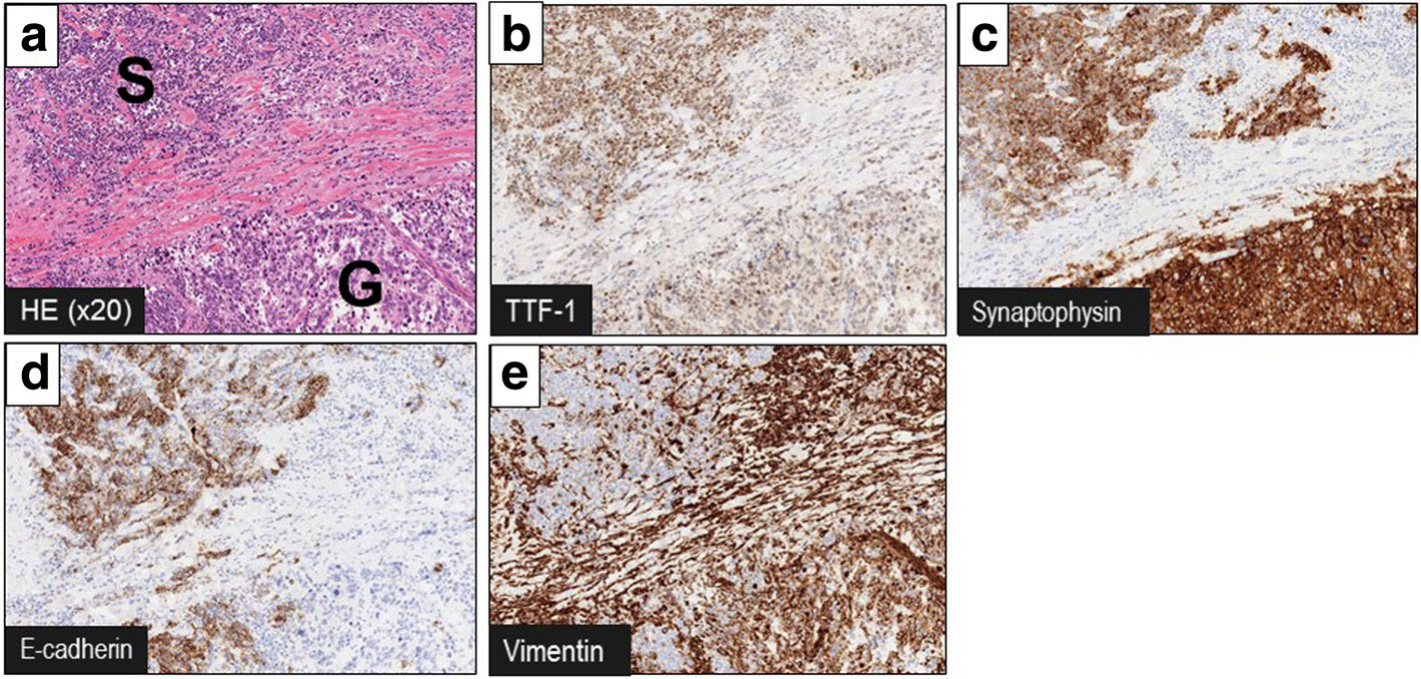
Immunohistochemical staining of the tumor. **a** Representative image of the tumor comprised of small cell component and giant cell component (hematoxylin and eosin stain). **b** Both the small cell and giant cell components were TTF-1-positive and p40-negative. **c** Both the small cell and giant cell components exhibited neuroendocrine differentiation (positive for synaptophysin and CD56 but negative for chromogranin A). **d**, **e** While the small cell component was E-cadherin-positive and vimentin-negative, the giant cell component was E-cadherin-negative and vimentin-positive, indicating EMT. *HE* hematoxylin and eosin, *G* giant cell component, *S* small cell component

Pathological investigation revealed a 22 × 18 × 12-mm nodule (Fig. 2a). Within the tumor, there was a dense sheet-like growth of small tumor cells with scant cytoplasm and finely granular nuclear chromatin (Fig. 2b–d). Moreover, there was a sheet-like growth of bizarre, highly pleomorphic mono- or occasionally multinucleated giant cells, accounting for approximately 40% of the tumor (Fig. 2c, e). Both the small and giant cell components were thyroid transcription factor 1 (TTF-1)-positive and p40-negative, exhibiting neuroendocrine differentiation, as indicated by positivity for synaptophysin and CD56 and negativity for chromogranin A (Fig. 3a–c). While the small cell component was E-cadherin-positive and vimentin-negative, the giant cell component was E-cadherin-negative and vimentin-positive, indicating epithelial-to-mesenchymal transition (EMT) (Fig. 3d, e). Only the small cell component was found within the mediastinal and hilar lymph nodes. The final pathological diagnosis was combined SCLC and GC, pT1bN2M0, pStage IIIA. The patient received adjuvant chemotherapy with 4 cycles of cisplatin and irinotecan. No sign of recurrence has been noted for 1 year after the surgery.

## Discussion

This is the first detailed report of a unique surgical case with combined SCLC and GC. The coexistence of SCLC and GC, epithelial and mesenchymal components, in the presented case might indicate several possibilities that should be investigated in further studies.

GC may arise from SCLC via EMT as SCLC progresses. Kuo et al. showed that normal pulmonary neuroendocrine cells are capable of EMT, sometimes called “slithering” during their migration [4]. The slithering program may contribute to EMT in SCLC, but the exact mechanism remains unclear. Reportedly, SCLC may be subdivided into two distinct classes based on its global gene expression pattern: “neuroendocrine” class and “mesenchymal-like” class, both of which can coexist [5]. The “mesenchymal-like” class SCLC might have the capacity of converting into GC, but this speculation should be further investigated.

Begin et al. found 4 autopsy cases of advanced SCLC combined with GC among 409 biopsy or autopsy cases during their 2-year study period [6]. They suggested that SCLC might develop into GC regardless of irradiation, because 3 of the 4 cases showed combined SCLC and GC in the non-irradiated metastatic lesions. Furthermore, Yamamoto and colleagues reported a rare case of esophageal GC combined with small cell carcinoma, suggesting the origin of GC may be associated with small cell carcinoma [7]. SCLC may arise from GC through phenotypic conversion. Approximately 15% of patients with lung adenocarcinoma undergoing epidermal growth factor receptor tyrosine kinase inhibitor treatment experience adenocarcinoma-to-SCLC conversion [8]. However, it is unclear whether GC-to-SCLC conversion can occur spontaneously with no treatment stimuli.

SCLC and GC may also be simultaneously derived from monoclonal or multiclonal cells of origin. Reportedly, SCLC develops from neuroendocrine precursors or alveolar type 2 cells [5], whereas GC develops from undifferentiated multipotent stem cells. However, it is still unknown whether these precursors can differentiate into two distinct tumor cell groups: SCLC and GC. Further investigation is necessary to reveal the underlying pathological process.

Our patient was treated with 4 cycles of cisplatin and irinotecan as postoperative chemotherapy. Accumulating evidence supports the idea that patients with completely resected pN2 SCLC or pN2 GC may benefit from adjuvant chemotherapy [9–12]. However, since there are only a few single-arm phase II studies evaluating adjuvant chemotherapy for SCLC and no prospective study investigating adjuvant chemotherapy for GC has been reported to date, the optimal regimen for pN2 combined SCLC and GC is unknown. Tsuchiya et al. reported from their phase II study of 62 patients that complete resection of pStage I to IIIA SCLC followed by adjuvant chemotherapy comprised of cisplatin and etoposide (PE) showed favorable outcomes [9]. Specifically, they reported a 5-year survival rate of 39% for pN2 SCLC. National Comprehensive Cancer Network guidelines recommend postoperative concurrent chemoradiotherapy for node-positive SCLC, but it is not routinely performed in Japan because local therapy is considered complete after surgery and also because a meta-analysis by Warde et al. showed that the addition of thoracic radiation therapy was associated with a 1.2% increased risk of treatment-related death [13]. Cisplatin and irinotecan (PI) is associated with better survival outcomes and less hematologic toxicity compared to the PE regimen in SCLC patients younger than 70 years who have extensive disease [14]. Additionally, PI has been shown to be effective for advanced non-small cell lung cancer [15, 16]. Based on these data along with the fact that the GC component could be a phenotype of NSCLC, a PI regimen was expected to be effective for both SCLC and GC components in the presented case.

## Conclusions

In conclusion, this is the first detailed report of a unique surgical case with combined SCLC and GC. The coexistence of SCLC and GC in the presented case might indicate several possibilities: (1) GC may arise from SCLC via epithelial-to-mesenchymal transition, (2) SCLC may arise from GC through phenotypic conversion, and (3) SCLC and GC may have derived from a common neuroendocrine origin. Further investigation is necessary to reveal the underlying pathological process and to establish the optimal treatment strategy.

## Abbreviations

CT: Computed tomography
EMT: Epithelial-to-mesenchymal transition
FDG: 18F-fluorodeoxyglucose
GC: Giant cell carcinoma
SCLC: Small cell lung carcinoma
